# Junge Erwachsene mit Autoinflammation – was kommt auf Rheumatolog:innen zu?

**DOI:** 10.1007/s00393-026-01871-9

**Published:** 2026-09-28

**Authors:** Marc-Alexander Oestreich, Jessica Pauz, Mary-Louise Daly, Martin Krusche, Tatjana Welzel

**Affiliations:** 1 Pädiatrische Rheumatologie, Universitäts-Kinderspital beider Basel (UKBB), Universität Basel, Basel, Schweiz; 2 Pädiatrische Rheumatologie, Universitätsklinik für Kinderheilkunde, Inselspital, Universitätsspital Bern, Universität Bern, Bern, Schweiz; 3 Medizinische Klinik und Poliklinik III, Sektion Rheumatologie und entzündliche Systemerkrankungen, Universitätsklinikum Hamburg-Eppendorf, Hamburg, Deutschland; 4 Rheumatologie, Universitätsspital Basel (USB), Universität Basel, Basel, Schweiz; 5 Pädiatrisches Forschungszentrum, Universitäts-Kinderspital beider Basel (UKBB), Universität Basel, Basel, Schweiz; 6 Deutsches Rheumaforschungszentrum Berlin, ein Leibniz-Institut, Programmbereich Epidemiologie, Berlin, Deutschland; 7 Pädiatrische Rheumatologie, Universitäts-Kinderspital beider Basel (UKBB), Spitalstrasse 33, 4031 Basel, Schweiz

**Keywords:** Autoinflammatorische Erkrankungen, Transition, Kinder und Jugendliche, Chronische Entzündung, Empowerment, Autoinflammatory diseases, Transition, Children and adolescents, Chronic inflammation, Empowerment

## Abstract

**Hintergrund:**

Fortschritte in Diagnostik, Molekulargenetik und zielgerichteten Therapien haben die Versorgung von Patient:innen mit autoinflammatorischen Erkrankungen (AID) optimiert. Dadurch erreichen mehr Kinder und Jugendliche mit AID das Erwachsenenalter, wodurch die Transition in die internistische Rheumatologie an Bedeutung gewinnt.

**Ziel der Arbeit:**

Darstellung der Besonderheiten der Transition von Jugendlichen mit AID von der pädiatrischen in die internistische Rheumatologie sowie Zusammenfassung aktueller Empfehlungen.

**Material und Methoden:**

Narrative Übersichtsarbeit auf Basis publizierter Daten (Leitlinien, Konsensuspapiere, Register‑, Kohortenstudien und Surveys).

**Ergebnisse:**

Die Transition von Jugendlichen mit AID erfordert einen frühzeitigen, strukturierten und multidisziplinären Prozess, der medizinische, psychosoziale und edukative Aspekte berücksichtigt, Selbstmanagement und Eigenverantwortung fördert sowie die professionelle Therapiekontinuität sichert. Empfehlungen für chronisch-entzündlich rheumatische Erkrankungen sind grundsätzlich auf AID übertragbar. AID weisen jedoch Besonderheiten wie genetische Ursachen, relevante Komorbiditäten und häufig lebenslange, teils Off-label-Therapien auf, die spezifische Anforderungen an den Transitionsprozess stellen. AID-spezifische Empfehlungen und Daten zur Wirksamkeit strukturierter Transitionsprogramme sind bislang begrenzt.

**Diskussion:**

Die Transition von Patient:innen mit AID ist anspruchsvoll. Eine enge Zusammenarbeit zwischen pädiatrischer und internistischer Rheumatologie sowie patientenzentrierte Transitionskonzepte sind entscheidend, um Krankheitskontrolle, Lebensqualität und gesellschaftliche Teilhabe zu sichern.

## Abstract (en)

**Background:**

Advances in diagnostics, molecular genetics and targeted treatment have optimized the management of patients with autoinflammatory diseases (AID). As increasing numbers of children and adolescents with AID survive into adulthood, the transition from pediatric to adult rheumatology care has become an important component.

**Aim of the study:**

Presentation of the specific challenges and requirements of the transition of adolescents with AID from pediatric to adult rheumatology care and summary of current evidence and recommendations.

**Material and methods:**

Narrative review of the literature including guidelines, consensus statements, registries, cohort studies and surveys addressing transition.

**Results:**

Successful transition of adolescents with AID requires an early structured and multidisciplinary process that addresses medical, psychosocial and educational needs, promotes self-management and empowerment and ensures continuity of specific care. Existing transition recommendations for chronic inflammatory rheumatic diseases are generally applicable to patients with AID; however, several AID-specific features necessitate additional considerations, such as genetic causes, relevant comorbidities and often lifelong treatment strategies involving off-label treatment. The AID-specific recommendations and data on the effectiveness of structured transition programs are so far limited.

**Discussion:**

The transition of patients with AIDs from pediatric to adult care is a complex and demanding process. Close collaboration between pediatric and adult rheumatologists as well as patient-centered transition strategies, are crucial to ensure disease control, quality of life and social participation.

Die Versorgung von Patient:innen mit autoinflammatorischen Erkrankungen („autoinflammatory diseases“; AID) hat sich durch Fortschritte in Diagnostik, Molekulargenetik und zielgerichteten Therapien deutlich verbessert. Dadurch erreichen zunehmend Kinder und Jugendliche das Erwachsenenalter, sodass die Transition in die internistische Rheumatologie zunehmend an Bedeutung gewinnt. Aufgrund der chronischen Krankheitslast, komplexer Therapieentscheidungen, Komorbiditäten sowie Fragen der Familienplanung ist eine strukturierte, interdisziplinäre Transition für eine kontinuierliche und hochwertige Versorgung essenziell.

## Autoinflammation – Herausforderungen für pädiatrische und internistische Rheumatologen

Autoinflammatorische Erkrankungen (AID) sind chronische seltene Erkrankungen, die durch Dysfunktion des angeborenen Immunsystems zu schubweiser oder dauerhafter Inflammation führen [2, 3]. Sie können mit einer Aktivierung des adaptiven Immunsystems einhergehen und sich mit erhöhter Suszeptibilität gegenüber Infektionen, Phänomenen von Autoimmunität oder unkontrollierter Hyperinflammation präsentieren [27]. Charakteristische und vielfach beschriebene klinische Symptome sind wiederkehrendes Fieber, Entzündungen im zentralen Nervensystem, der (Sinnes‑)Organe, muskuloskeletal und (muko)kutan, meist gekoppelt mit laborchemischer Inflammation. Durch unkontrollierte Krankheitsaktivität können AID zu reduzierter Lebensqualität und zu Morbidität sowie mitunter zu potenziell verfrühter Mortalität führen [25]. Auch besteht ein erhöhtes Risiko für soziökonomische Herausforderungen und psychische Begleiterkrankungen bei den Betroffenen und ihren Familien [13, 18].

**Abb. 1:**
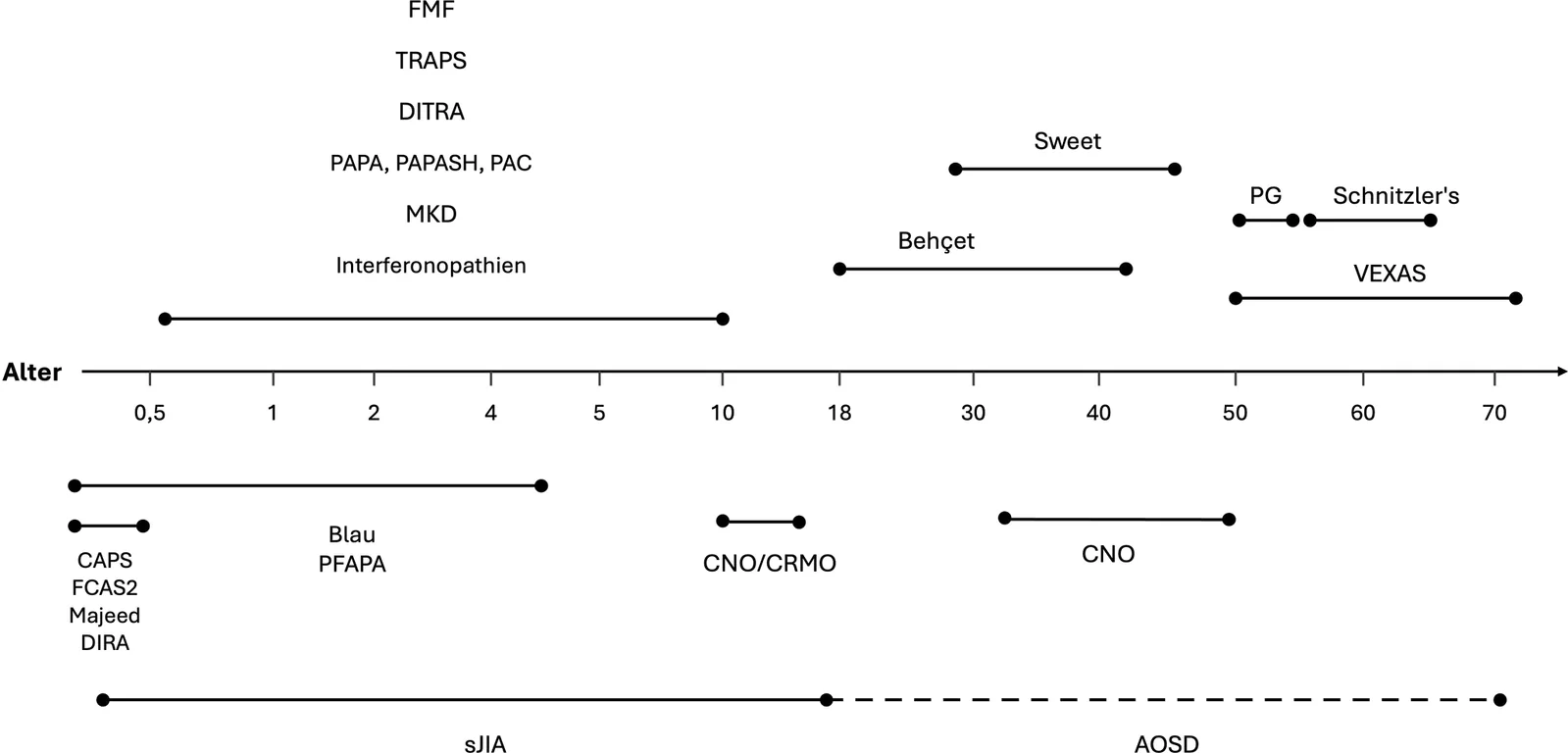
Typisches Manifestationsalter für ausgewählte autoinflammatorischen Erkrankungen. *FMF* familiäres Mittelmeerfieber, *TRAPS* TNF-Rezeptor-assoziiertes periodisches Syndrom, *DITRA* Defizienz des Interleukin-36-Rezeptor-Antagonisten, *PAPA* pyogene Arthritis, Pyoderma gangraenosum und Akne, *PAPASH* pyogene Arthritis, Pyoderma gangraenosum, Akne und Hidradenitis suppurativa, *PAC* Pyoderma gangraenosum, Akne und Colitis ulcerosa, *MKD* Mevalonatkinasedefizienz, *VEXAS* VEXAS-Syndrom (Vakuolen, E1-Enzym, X‑chromosomal, autoinflammatorisch, somatisch), *PFAPA* periodisches Fieber mit Aphthen, Pharyngitis und Adenitis, *CNO* chronisch-nichtbakterielle Osteomyelitis, *CMRO* chronisch-rekrutierende multifokale Osteomyelitis, *sJIA* systemische juvenile idiopathische Arthritis, *AOSD* Morbus Still des Erwachsenen (Adult-onset Still’s Disease), *CAPS* Cryopyrin-assoziierte periodische Syndrome, *FCAS2* familiäres kälteinduziertes autoinflammatorisches Syndrom Typ 2, *DIRA* Defizienz des Interleukin-1-Rezeptorantagonisten. (Mod. nach [1])

Der kontinuierliche molekulargenetische und translationale Fortschritt in den letzten Jahren hat zur Beschreibung zunehmender AID-Entitäten geführt, wobei einige eher im Kindesalter, andere eher im Erwachsenenalter manifest werden können (Abb. 1). Das stetig zunehmende Wissen um pathogenetisch relevante Signalwege, aber auch die Einführung von auf Next-Generation Sequencing (NGS) basierten Verfahren und funktioneller Teste ermöglichte die Identifikation komplexerer Vererbungsmechanismen sowie Detektion von somatischen Mosaiken, bei denen der Grad der Mosaike mit dem Alter bei Symptombeginn und der Krankheitsschwere zu korrelieren scheint [17].

Eine frühe Diagnosestellung ermöglicht AID-Patient:innen Zugang zu wirksamen Therapien

Diese Fortschritte ermöglichten es, in den letzten Jahren eine wachsende Anzahl an Kindern und Jugendlichen mit einer AID zu diagnostizieren. Für viele seit Längerem beschriebene monogenetischen AID hat sich die mediane Zeit bis zur Diagnosestellung in den letzten Jahren deutlich reduziert (Median: 1,2 Jahre) [26]. Eine wichtige Determinante für die Dauer bis zur Diagnosestellung scheint dabei die Familienanamnese. Ist bei einem jungen Erwachsenen eine AID bekannt, kann dies Auswirkungen auf die Familienplanung, die Schwangerschaft sowie die postpartale und neonatale Diagnostik und Therapie haben.

Eine frühe Diagnosestellung ermöglicht vielen AID-Patient:innen einen Zugang zu einer wirksamen Therapieoption, z. B. in Form von steroidsparenden Therapien mit Colchicin, Interleukin(IL)-1/IL-6-Inhibitoren, Januskinase(JAK)-Inhibitoren oder Tumornekrosefaktor(TNF)-Inhibitoren. Gleichzeitig bleibt die Behandlung komplex, insbesondere bei unklassifizierten AID; zudem ist häufig eine Off-label-Therapie erforderlich. Ziel der Behandlung ist das Erreichen einer vollständigen Krankheitsaktivitätskontrolle oder einer minimalen Krankheitsaktivität [4, 7, 12, 19, 23]. Validierte Instrumente wie der Patient-Reported Autoinflammatory Disease Activity Index (AIDAI) und der Autoinflammatory Disease Damage Index (ADDI) unterstützen bei der Quantifizierung von entzündlicher Aktivität und Schäden, weshalb sie für das Langzeit-Monitoring und kontinuierliche Therapieanpassungen essenziell sind [21, 25].

Eine autoinflammatorische Erkrankung hat je nach Lebensphase unterschiedliche Auswirkungen auf Betroffene, Familienangehörige und potenziellen Nachwuchs. Dies unterstreicht angesichts der zunehmenden Zahl jugendlicher Patient mit AID die Bedeutung einer engen Zusammenarbeit zwischen der pädiatrischen und internistischen Rheumatologie und die Notwendigkeit einer strukturierten Transition [22]. Aufgrund der zunehmenden Lebenserwartung vieler Patient:innen rücken neben der Sicherstellung einer kontinuierlichen Versorgung insbesondere die langfristige Kontrolle der Krankheitsaktivität durch personalisierte Therapien und Off-label-Ansätze, regelmäßiges klinisches Monitoring (einschließlich AIDAI und ADDI), Labor- und Organkontrollen sowie die Familienplanung und das Management von Komorbiditäten zunehmend in den Fokus.

## Transition und AID

Die Transition fällt für die jungen Patient:innen mit AID in eine wichtige Lebensphase, die den Übergang in die Selbstständigkeit und Eigenverantwortlichkeit sowie andere wichtige Entwicklungsschritte mit sich bringt. Integrale Bestandteile der Transition sind daher neben medizinischen Aspekten auch psychische Gesundheit, das Körperbild, die Frage nach der Offenlegung der Erkrankung und soziale Determinanten wie Bewegungsverhalten, Noxenkonsum, sexuelle Gesundheit, kulturelle Besonderheiten, Bildungs- und Berufsfragen sowie berufliche Vorbereitung einschließlich Praktika, Kompetenzerwerb, Kenntnis von Rechten und Ressourcen [28]. Schwankende Krankheitsaktivität und eine unzureichende Krankheitskontrolle können die Transition zusätzlich erschweren. Da ein Zusammenhang zwischen psychischen Erkrankungen und AID-Aktivität bei jungen Menschen zu bestehen scheint, müssen mögliche Angststörungen und Depressionen in Abhängigkeit zur Krankheitsdauer, Symptomschwere und Auftreten sowie Schubhäufigkeit und Therapieansprechen frühzeitig bedacht werden [6]. Eine strukturierte Vorbereitung auf den Übergang von einer eher familienzentrierten pädiatrischen Versorgung in die stärker auf Eigenverantwortung und Selbstmanagement ausgerichtete Erwachsenenrheumatologie ist daher zentral für das weitere Management der AID.

Eine alters- und entwicklungsadäquate Versorgung über die gesamte Lebensspanne ist bedeutend

Die Empfehlungen der Fachgesellschaften European Alliance of Associations for Rheumatology (EULAR) und Paediatric Rheumatology European Society (PreS) zur transitionalen Versorgung junger Menschen mit pädiatrisch-rheumatologischen Erkrankungen beschreiben generische, krankheitsübergreifende Qualitätsindikatoren. Dazu gehören ein früh beginnender, multidisziplinärer Prozess, eine definierte Transitionskoordination, klare Übergabedokumente und eine gesicherte Therapiekontinuität [10]. Die Empfehlungen des französischen FAI2R-Netzwerks ergänzen eine früh beginnende, schrittweise Übertragung von Eigenverantwortung, ein gemeinsames Transitionsprotokoll sowie therapeutische Schulungsprogramme als zentrale Erfolgsfaktoren [11]. Viele dieser Empfehlungen können bei AID herangezogen werden. Allerdings muss bedacht werden, dass AID zumeist genetisch bedingt sind, chronisch verlaufen, vielfach einer lebenslangen Therapie und eines multidisziplinären Managements bedürfen. Neben Krankheits- und Therapiekomplikationen sind durch die AID verursachte körperliche Beeinträchtigungen (z. B. Hautveränderungen, Arthritis, Schwerhörigkeit, Visusproblematiken, Knochendeformitäten) mit Auswirkung auf das Erscheinungsbild möglich. Isolation, Verlust des Selbstvertrauens, Angst und Depression sind bei AID nicht selten, und regelmäßige Krankenhausbesuche, Schmerzen und Fatigue beeinträchtigen die Lebensqualität der jungen Erwachsenen. Hinzu kommen Beziehungsprobleme und Herausforderungen in der Familie sowie Unverständnis in der Schule oder beim Arbeitgeber [11]. Eine alters- und entwicklungsadäquate Versorgung über die gesamte Lebensspanne hinweg mit Förderung des Selbstmanagements, der partizipativen Entscheidungsfindung, reproduktiver Gesundheit mit spezialisierte AID-Transitionssprechstunden sind daher bedeutend [23]. Daten zum Transitionsprozess bei AID, der Wirksamkeit und Besonderheiten sind jedoch bisher noch limitiert.

### Infobox Rolle und mögliche Aufgaben einer Transition Nurse

Eine Transition Nurse begleitet Jugendliche beim Übergang von der Kinder- in die Erwachsenenklinik. Sie lernt idealerweise die Jugendlichen in der Kinderklinik kennen und begleitet sie auch in der Erwachsenenklinik weiter. Sie bietet Kontinuität und unterstützt beim Kennenlernen des neuen Behandlungsteams, hilft die Erkrankung und die Therapie besser zu verstehen und fördert Selbstmanagement-Kompetenzen, so dass die Jugendlichen zunehmend selbstständig Verantwortung für ihre Gesundheit übernehmen können. Mögliche Aufgaben sind: Medikamentenschulungen, Telefonate mit Jugendlichen zur Triage/Beratung von Anliegen, Unterstützung in der Schule und dem Ausbildungsbetrieb (z. B. Nachteilsausgleich, Sportbefreiung), Koordination von Behandlungsterminen sowie Umorganisation.

### Spezifische Empfehlungen und Erkenntnisse

Eine europaweite Erhebung der Arbeitsgruppe „Transition“ des Referenznetzwerks für Immundefekte, autoinflammatorische und Autoimmunerkrankungen ergab, dass alle teilnehmenden Zentren Patienten in die Erwachsenenversorgung überführen. Der Prozess zeigte sich jedoch hinsichtlich des Beginns (16–18 Jahre), des Übergabealters (18–20 Jahre), der Struktur und der Dokumentation variabel. Obwohl etwa 75 % der primären Immundefizienz- und AID-Zentren über einen festgelegten Transitionsprozess berichteten, verfügten nur wenige über nationale oder krankheitsspezifische Leitlinien [16]. In 2024 verfasste diese Arbeitsgruppe umfassende, evidenzbasierte Konsensus-Empfehlungen für die Transition von Patient:innen mit primären Immundefekten und AID. Die Empfehlungen adressieren die Struktur und Administration von Transitionsprogrammen, die Zusammenarbeit mit sozialen Systemen (Tab. 1) sowie Kontraindikationen für eine Transition [15]. Vor der Übergabe sollten diagnostische Befunde und Managementpläne sowie multidisziplinäre Nachsorge vereinbart werden. Alle relevanten Unterlagen, einschließlich genetischer Befunde, Informationen zu Studienbeteiligungen und Nachsorgeplänen, sollten vor dem ersten Termin in der Erwachsenenversorgung übermittelt werden [15]. Empfohlen werden die interdisziplinäre Einbindung von Patient:innen, Bezugspersonen mit Fachkräften und Study Nurses sowie gemeinsame pädiatrisch-internistische Übergangssprechstunden. Standardisierte Assessments zur Transitionsbereitschaft sollen die Grundlage für altersgerechte Versorgungspläne für medizinische, psychosoziale, schulische und berufliche Belange bilden. Schulungen zu Krankheitsnatur, Therapieoptionen, Medikamenten-Selbstapplikation und Schub-Management sollen ebenso erfolgen wie die Vermittlung von Informationen zu Patientenrechten und Nachteilsausgleichen [15]. Die am Transitionsprozess Beteiligten sollten mit der Lebenssituation und der AID-Erkrankung des Patienten vertraut sein und regelmäßig auf den neuesten Stand gebracht werden [22]. Ein Transitionsaufschub wird bei kritischer Erkrankung, instabiler Krankheitslage, fehlender Versorgungsinfrastruktur im Erwachsenenbereich oder fehlender Kontinuität bei laufenden klinischen Studien empfohlen; vor dem Transfer sollte eine klinische Stabilisierung erfolgen [15]. Aktuell verfügbare Daten zu Transitionseffekten bei AID sind begrenzt, deuten aber bereits an, dass strukturierte, AID-spezifische Transitionsprogramme die Versorgungskontinuität verbessern. So konnte in einer retrospektiven monozentrischen Kohortenstudie mit 111 jungen Erwachsenen mit AID gezeigt werden, dass eine formal durchgeführte, strukturierte Transition mit einer signifikant höheren Rate an fortgesetzter spezialisierter Betreuung assoziiert war [8]. Die Art der Transition (Überweisungsschreiben vs. pädiatrisch-internistische Konsultation) zeigte in dieser Studie keinen signifikanten Einfluss auf die Kontrolle der Krankheitsaktivität. Zur Förderung der aktiven Beteiligung und des Selbstmanagements ist ein kooperativer Prozess mit gemeinsamen Konsultationen sinnvoll, auch wenn die strukturierte Transition zeit- und ressourcenintensiv ist [5]. So lag in einer prospektiven deutschen Kohortenstudie mit 58 Jugendlichen die mittlere Prozessdauer von der Entscheidung zur Transition bis zur Übergabe bei 315 ± 147 Tagen, war aber mit einer hohen Erfolgsquote (84,5 % abgeschlossene Übergaben) verbunden [5]. Die meisten Termine wurden interdisziplinär mit im Median 3 Teammitgliedern durchgeführt und dauerten 65,5 ± 21,3 min [5].

**Tab. 1:** Empfehlungen modifiziert nach Israni et al. 2025. *ADDI* Autoinflammatory Disease Damage Index

Dimension	Ziel	Empfehlungen
1. Planung und Organisation	Strukturierte und frühzeitige Vorbereitung der Transition	Transitionspartner und integrierte Versorgungswege definieren
		Transitionsprotokoll erstellen
		Zuständigkeiten und (Haupt‑)Ansprechpartner festlegen
		Planung möglichst vor dem 16. Lebensjahr für die Patient:innen beginnen
		Transitionskoordinator benennen
		Versorgung an klinischen und persönlichen Bedürfnissen anpassen
	Interdisziplinäre Zusammenarbeit und Kommunikation sicherstellen	Multidisziplinäre Fallbesprechungen und Versorgung etablieren
		Personal zu Adoleszenz und Transition schulen
		Effektive Kommunikation sicherstellen
		Patientenakte und relevante Unterlagen rechtzeitig übermitteln
		Erreichbarkeit (Telefon/E-Mail) und Zuständigkeit klären sowie gewährleisten und an Patient:innen kommunizieren
2. Struktur und Ablauf der Transition	Kontinuierlichen und koordinierten Übergang gewährleisten	Gemeinsame Transitionssprechstunde etablieren
		Gemeinsame Nachsorge im Jahr vor dem Transfer
		Pädiatrische Versorgungskontinuität bis zur Erstvorstellung in der Erwachsenenrheumatologie sicherstellen
	Individuellen Übergangs- und Versorgungsplan erstellen	Personalisierter Übergangsplan
		Aktive und inaktive Problemliste sowie Follow-up dokumentieren
		Medikation und Behandlungsplan prüfen
		(Virtuelle) Besichtigung der internistischen Abteilung
		Termine schul- und alltagsfreundlich planen
3. Patientenzentrierte Transition	Selbstständigkeit und aktive Beteiligung fördern	Jugendliche aktiv einbinden
		Elternbeteiligung schrittweise reduzieren
		Eigenverantwortliches Management (Symptome, Medikation, Termine) fördern
		Shared Decision-Making fördern
		Zeit für Fragen einplanen
	Individuelle und soziale Bedürfnisse berücksichtigen	Funktionsstatus und psychosoziale Situation standardisiert erfassen
		Psychologische Unterstützung anbieten
		Ganzheitliche Versorgung (psychosoziale/berufliche Aspekte) sicherstellen
		Bildungs- und Berufsberatung anbieten
	Adhärenz und langfristige Selbstversorgung unterstützen	Therapieadhärenz überprüfen
		Beratung bei Non-Compliance
		Altersgerechte hausärztliche Versorgung sicherstellen
		Zugang zu lokalen Langzeittherapien gewährleisten
4. Medizinische und ergänzende Aspekte	Umfassende medizinische Versorgung sicherstellen	Alle Organmanifestationen berücksichtigen
		Diagnostische Reevaluation bei unklarer Diagnose
		Strukturiertes Monitoring gewährleisten (z. B. Organscreening, ADDI)
		Medikationsfortführung bestätigen
		Impfstatus dokumentieren bzw. Impfplan für das Erwachsenenalter erstellen
	Patient und Umfeld informieren und unterstützen	Mündliche und schriftliche Information zu Erkrankung, Genetik, Therapie und Schüben
		Auf verlässliche Internetseiten hinweisen
		Einwilligung zur Informationsweitergabe einholen
		Bei Bedarf Schule oder Arbeitsplatz über Erkrankung und Termine informieren
		Peer-Kontakte und Patientenvertretungen fördern
		Auf Netzwerke lokal, (inter)national hinweisen
		Über Patientenrechte landesspezifisch informieren
5. Forschung und Studien	Kontinuität der Forschungsbeteiligung gewährleisten	Studienunterlagen an das weiterbehandelnde Team übermitteln
		Studienstatus und Nachsorgepläne in der Patientenakte dokumentieren
		Kontinuität des Studienmonitorings sicherstellen
		Studienergebnisse an das Ursprungsteam zurückmelden
	Studienbezogene Anforderungen im Transitionsprozess	Bei Bedarf erneute Studienaufklärung und Einwilligung einholen und dokumentieren

## Ausgewählte Themen in und während der Transition

### Therapie

Therapeutische Strategien sollten im jungen Erwachsenenalter überprüft werden, da insbesondere in der Pädiatrie ein hoher Off-label-Einsatz bei seltenen und unklassifizierten AID notwendig ist. Dies sollte unter Berücksichtigung von Krankheitsaktivität, krankheitsassoziiertem Schaden, Leitlinienempfehlungen, Indikation, Nutzen-Risiko-Profil, Zulassungsstatus, Erstattung sowie Aufklärungs- und Dokumentationspflichten erfolgen. Analog zur pädiatrischen Rheumatologie sollte während und nach dem Transitionsprozess die standardisierte Verlaufsbeurteilung und systematische Erfassung der Krankheitsaktivität und krankheitsassoziierter Schäden, z. B. mithilfe des AIDAI und ADDI, weiterhin erfolgen.

### Adhärenz

Gründe für Non-Adhärenz gegenüber der Medikation können mangelndes Verständnis und AID-Priorisierung, unerwünschte Ereignisse, negative Auswirkungen auf Sozialisierung und Beziehungen sowie die parenterale Verabreichung von Biologika sein [22]. Zusätzlich können Trypanophobie (Nadelfurcht) und Abneigung gegenüber Selbstverabreichung bei Jugendlichen zur Non-Adhärenz beitragen. Informationen über Folgen einer Non-Adhärenz können den Jugendlichen dazu motivieren, Verantwortung für sich zu übernehmen und an einer gemeinsamen Entscheidungsfindung zu beteiligen [22]. Ebenso wird das Erkennen von Schub-Triggern (z. B. Kälte, Überanstrengung, Schlafmangel) dabei helfen, diese zu vermeiden oder angemessen damit umzugehen. Eine Therapieadhärenz ist wichtig, um irreversible Organschäden zu verhindern [19].

### Sexualität und Familienplanung

Im jungen Erwachsenenalter gewinnen Themen wie Sexualität und Familienplanung an Bedeutung. Bei konkretem Kinderwunsch sollte eine strukturierte Beratung zur individuellen Risikokonstellation, dem Stellenwert von Krankheitsaktivität und Therapieadhärenz sowie ggf. eine genetische Beratung erfolgen. Die verfügbaren Daten zu Schwangerschaften bei AID sind aufgrund der Seltenheit und Heterogenität der Erkrankungen begrenzt. Schwangerschaftsverlauf und Komplikationsrisiko werden wesentlich durch Krankheitsaktivität, Organschäden und Therapie beeinflusst. Bei guter präkonzeptioneller Krankheitskontrolle und schwangerschaftskompatibler Medikation sind für viele Patientinnen günstige Verläufe möglich. Eine persistierende systemische Inflammation kann jedoch mit einem erhöhten Risiko für Schwangerschaftskomplikationen verbunden sein. Für das FMF liegt die umfangreichste Datenbasis vor: Eine aktuelle Metaanalyse zeigte insbesondere eine erhöhte Rate an Frühgeburtlichkeit (8,7 % vs. 5,4 %; Odds-Ratio 1,67; 95 % Konfidenzintervall [KI] 1,05–2,67). Für fetale Wachstumsrestriktion und hypertensive Schwangerschaftserkrankungen fanden sich ebenfalls numerisch erhöhte Raten, die jedoch keine statistische Signifikanz erreichten [14]. Daher wird eine interdisziplinäre und engmaschige Betreuung während der Schwangerschaft empfohlen. Für suffizient behandelte FMF-Patient:innen zeigen die verfügbaren Daten keine relevante Beeinträchtigung der Fertilität [24]: Rezidivierende peritoneale oder pelvine Entzündungen können jedoch Adhäsionen und Tubenverschlüsse begünstigen. Bei rezidivierenden skrotalen Beschwerden oder ungeklärter männlicher Infertilität sollte eine urologische Abklärung einschließlich Hormonanalyse und Spermiogramm erfolgen. Der Einfluss einer Schwangerschaft auf die Schubfrequenz ist variabel. Beschrieben wurden leicht erhöhte Raten von Frühgeburten sowie möglicherweise von fetaler Wachstumsrestriktion und hypertensiven Schwangerschaftserkrankungen. Ob peritonitische Attacken unmittelbar Fehlgeburten oder vorzeitige Wehen auslösen, ist nicht ausreichend belegt [20]. Colchicin weist ein günstiges Sicherheitsprofil ohne Hinweise für einen negativen Einfluss auf Schwangerschaftsverlauf oder neonatales Outcome auf. Bei entsprechender Indikation, insbesondere beim FMF, wird die konsequente Fortführung während Konzeption, Schwangerschaft und Stillzeit daher empfohlen. Auch IL-1-Inhibitoren, insbesondere Anakinra und Canakinumab [19], können bei relevanter Krankheitsaktivität und individueller Nutzen-Risiko-Abwägung in der Schwangerschaft eingesetzt werden [9].

### Impfungen und Reisen

Im Rahmen des Transitionsprozesses sollte der Impfstatus nochmals systematisch überprüft und bei Bedarf entsprechend aktuellen Empfehlungen (https://www.rki.de/DE/Themen/Infektionskrankheiten/Impfen/Staendige-Impfkommission/Immundefizienz/immundefizienz-node.html) vervollständigt werden. Da Impfungen bei AID vorübergehend inflammatorische Symptome oder Schübe triggern können, empfiehlt sich eine Planung in stabiler Krankheitsphase. Gleichzeitig sollten die Jugendlichen und jungen Erwachsenen auf der Grundlage ihrer AID und Therapie hinsichtlich Schutzimpfungen (z. B. Influenza-Impfungen) und allgemeinen Impfprinzipien (Tot- und Lebendimpfstoffe) beraten und informiert werden. Wichtig ist auch, Aspekte im Zusammenhang mit Reisen und Auslandsaufenthalten zu besprechen (z. B. Schutzimpfungen, Medikamentenmitnahme, Flugzertifikate, Notfallmedikamente, medizinische Versorgung).

## Nach der Transition: Komplikationen und Komorbiditäten

Die AA-Amyloidose, auch sekundäre Amyloidose, ist eine gefürchtete Komplikation in der internistischen Rheumatologie, für die das Risiko mit Dauer, genetischem Phänotyp sowie Ausmaß der ungenügend kontrollierten (sub)klinischen AID-Aktivität ansteigt. Insbesondere bei Hochrisikogenotypen sollten Entzündungsparameter, insbesondere C‑reaktives Protein (CRP) und Serum-Amyloid A sowie Nierenfunktion und Proteinurie engmaschig überwacht werden. Eine konsequente, Therapieadhärenz ist essenziell, um irreversible Organschäden und/oder einen Progress der Amyloidose zu verhindern.

## Fazit für die Praxis

- Autoinflammatorische Erkrankungen (AID) sind seltene, meist lebenslang verlaufende Erkrankungen, deren Diagnostik und Therapie sich durch molekulargenetische Fortschritte und zielgerichtete Behandlungsoptionen deutlich verbessert haben.
- Dadurch erreichen zunehmend Jugendliche mit AID das Erwachsenenalter, was eine strukturierte Transition in die internistische Rheumatologie erforderlich macht.
- Ziel ist die Sicherstellung einer kontinuierlichen Krankheitskontrolle, die Vermeidung von Organschäden sowie die Erhaltung von Lebensqualität und gesellschaftlicher Teilhabe.
- Neben medizinischen Aspekten müssen psychosoziale Herausforderungen, Selbstmanagement, Familienplanung und berufliche Perspektiven berücksichtigt werden.
- Frühzeitige Vorbereitung, standardisierte Übergabeprozesse, interdisziplinäre Zusammenarbeit und regelmäßiges Monitoring sind zentrale Erfolgsfaktoren, um Versorgungslücken zu vermeiden und die langfristige Betreuung von Patient:innen mit AID zu optimieren.

## Data Availability

Alle dieser Arbeit zugrunde liegenden Daten sind in diesem Artikel enthalten.
